# Identification of a Necroptosis-Related Prognostic Signature and Associated Regulatory Axis in Lung Adenocarcinoma

**DOI:** 10.1155/2023/8766311

**Published:** 2023-02-22

**Authors:** Libo Sun, Wenwen Li, Zhenhuan Zhao, Yanhua Zuo, Zhiwu Han

**Affiliations:** ^1^Department of Pharmacy, The Affiliated Hospital of Qingdao University, Qingdao, China; ^2^Department of Hematology, Qingdao Women and children's Hospital, Qingdao, China

## Abstract

**Background:**

Lung cancer is considered to be the second most aggressive and rapidly fatal cancer after breast cancer. Necroptosis, a novel discovered pattern of cell death, is mediated by Receptor-interacting serine/threonine-protein kinase 1 (RIPK1), Receptor-interacting serine/threonine-protein kinase 3 (RIPK3), and Mixed Lineage Kinase Domain Like Pseudokinase (MLKL).

**Methods:**

For the purpose of developing a prognostic model, Least absolute shrinkage and selection operator (LASSO) Cox regression analysis was conducted. Using Pearson's correlation analysis, we evaluated the correlation between necroptosis-related markers and tumor immune infiltration. A bioinformatics analysis was conducted to construct a necroptosis-related regulatory axis.

**Results:**

There was a downregulation of most of necroptosis-related genes in lung adenocarcinoma (LUAD) versus lung tissues but an increase in PGAM5, HMGB1, TRAF2, EZH2 levels. We also summarized the Single Nucleotide Variant (SNV) and copy number variation (CNV) of necroptosis-related genes in LUAD. Consensus clustering identified two clusters in LUAD with distinct immune cell infiltration and ESTIMATEScore. Genes related to necroptosis were associated with necroptosis, Tumor necrosis factor (TNF) signaling pathway, and apoptosis according to Gene Ontology (GO) and Kyoto Encyclopedia of Genes and Genomes (KEGG) pathways. Four prognostic genes (ALDH2, HMGB1, NDRG2, TLR2) were combined to develop a prognostic gene signature for LUAD patients, which was highly accurate in predicting prognosis. Univariate and multivariate analysis identified HMGB1, pT stage, and pN stage as independent factors impacting on LUAD patients' prognosis. A significant correlation was found between the level of TLR2 and NDRG2 and clinical stage, immunity infiltration, and drug resistance. Additionally, the progression of LUAD might be regulated by lncRNA C5orf64/miR-582-5p/NDRG2/TLR2.

**Conclusion:**

The current bioinformatics analysis identified a necroptosis-related prognostic signature for LUAD and their relation to immunity infiltration. This result requires further investigation.

## 1. Introduction

Lung cancer will account for d 228,820 new cases and 135,720 deaths in 2020 all globally [1]. Adenocarcinoma of the lung (LUAD) accounts for more than 40% of all cases of lung cancer [2]. Due to the absence of typical clinical symptoms, patients usually have disseminated metastatic tumors when initially diagnosed with LUAD. Worse still, LUAD is characterized by high aggression and rapidly fatality with overall survival (OS) less than 3 years [3]. Even though smoking has been identified as a risk factor for LUAD, the molecular mechanism is still not understood. And exploration and identification of the potential tumorigenesis molecular mechanism and prognostic markers for LUAD are urgent.

Necroptosis, a novel discovered pattern of cell death, is mediated by Receptor-interacting serine/threonine-protein kinase 1 (RIPK1), Receptor-interacting serine/threonine-protein kinase 3 (RIPK3), and Mixed Lineage Kinase Domain Like Pseudokinase (MLKL) [4, 5]. Studies have implicated necroptosis in the pathogenesis of Parkinson, Alzheimer, vascular atherosclerosis, and infectious disease [5–7]. The death of T cells and cancer metastasis can also be accelerated by necroptosis, according to recent evidence [8]. On the other hand, when immunotherapy is used to treat malignancies, necroptosis may trigger and amplify antitumor immunity [4]. Necroptosis regulators may also provide prognostic information for cancer and other diseases [9, 10]. Moreover, some necroptosis-related prognostic signature had been identified for types of cancer, including stomach adenocarcinoma, breast cancer and cervical squamous cell carcinoma, and endocervical adenocarcinoma [11–13]. As such, necroptosis-related genes may also contribute to LUAD prognosis.

Big data mining has been proposed as a promising tool for examining tumorigenesis mechanisms, associated prognosis markers, and therapy targets, following the development of the Cancer Genome Atlas (TCGA). Herein, we systematically investigated the expression of genes associated with necroptosis, their prognostic significance, and their association with immune infiltration. The data may offer another evidence about the vital functions of necroptosis in LUAD.

## 2. Materials and Methods

### 2.1. Datasets and Preprocessing

The RNA sequencing data (Fragments Per Kilobase of exon model per Million mapped fragments (FPKM) value) and copy number variation (CNV) data of LUAD patients were downloaded from TCGA database. After requiring the clinic characters of LUAD cohort from TCGA, we rearranged it and Supplementary Table 1 showed the detail. Using R (version 4.0.5) and R Bioconductor packages, dataset processing and further analysis were carried out. All the patients losing some clinical information were rejected and a total of 486 cases were obtained. In order to analyze the expression profile, we normalized it to transcripts per kilobase million values.

### 2.2. Expression, Genetic Mutation, and Functional Enrichment Analysis

By reviewing the previous literatures, we identified 17 necroptosis-related genes, including RIPK1, RIPK3, MLKL, TLR2, TLR3, TLR4, TNFRSF1A, PGAM5, ZBP1, NR2C2, HMGB1, CXCL1, USP22, TRAF2, ALDH2, EZH2, and NDRG2 [14–22]. Using “limma” and “reshape2” package [23], the expression patterns of genes associated with necroptosis were generated. A mutation frequency of the gene was calculated using “maftools” package [24]. The “RCircos” package also allowed us to visualize the chromosome locations of CNVs associated with necroptosis [25]. We then performed functional enrichment analysis (Gene Ontology (GO) and Kyoto Encyclopedia of Genes and Genomes (KEGG)) with “clusterProfiler “package [26].

### 2.3. Consensus Cluster Analysis

We then conducted consensus cluster analysis using the “ConsensusClusterPlus” package, which was calculated 1,000 times [27]. In the following step, survival and gene expression were analyzed using the packages “survival” and “pheatmap”. ESTIMATE algorithm was applied for evaluating the difference of Immunoscore, StromaScore, ESTIMATEScore, and immune cell infiltration in each cluster of LUAD. The immune cell landscape in each cluster of LUAD was generated with “vioplot” package.

### 2.4. Development of Necroptosis-Related Prognostic Gene Signature

The log-rank test was used to calculate the *p*-values, and hazard ratio for the necroptosis-related prognostic gene found by Kaplan–Meier analysis. In the following step, the development of the prognostic model was counted on the Least absolute shrinkage and selection operator (LASSO) Cox regression analysis based on necroptosis-related prognostic genes. The risk score of each LUAD patient was calculated by a computational equation (sum of coefficients × necroptosis-related gene expression). TCGA-LUAD patients were separated into two subgroups (low- and high-risk), and the cut-off was the median value of the gene expression. We used Kaplan-Meier analysis to determine the OS curve, and time ROC analysis to determine how well this prognostic signature predicted outcomes. The prognostic risk factor was further analyzed using univariate and multivariate Cox analysis using clinical characteristics and gene signatures. A predicted nomogram was developed to evaluate the predictive performance in OS rate (1-, 3-, and 5-year).

### 2.5. Prognostic Gene Individual Analysis

Wilcox test was utilized to evaluated expression difference of prognostic signature in different pathological stage of LUAD patients. A correlation was then determined between prognostic signature and immune cell infiltration using Tumor Immune Estimation Resource (TIMER) (https://cistrome.shinyapps.io/timer/). This was followed by tumor mutation burden (TMB) and microsatellite instability (MSI) analysis using Spearman's correlation method. We then collected the IC50 of 265 small molecules in 860 cell lines, and its corresponding necroptosis-related prognostic gene expression from Genomics of Drug Sensitivity in Cancer (GDSC). Using Pearson correlation analysis, the relation between gene expression and drug IC50 was determined.

### 2.6. lncRNA–miRNA–MRNA Network Construction

The miRNA targets of necroptosis-related prognostic gene were predicted with miRDB (http://mirdb.org/) [28]. Moreover, we evaluated the expression and prognosis significance of these miRNAs in LUAD to further screen the most promising targets. This was followed by lncRNA targets predicting using LncBase (https://carolina.imis.athena-innovation.gr/) [29] and RNAInter (http://www.rna-society.org/) [30] with a coefficient>0.7. A similar approach to identifying promising lncRNA targets was used to investigate the expression and prognosis significance of these lncRNAs.

## 3. Results

### 3.1. Expression and Mutation Landscape of Necroptosis-Related Genes in LUAD

**Figure 1:**
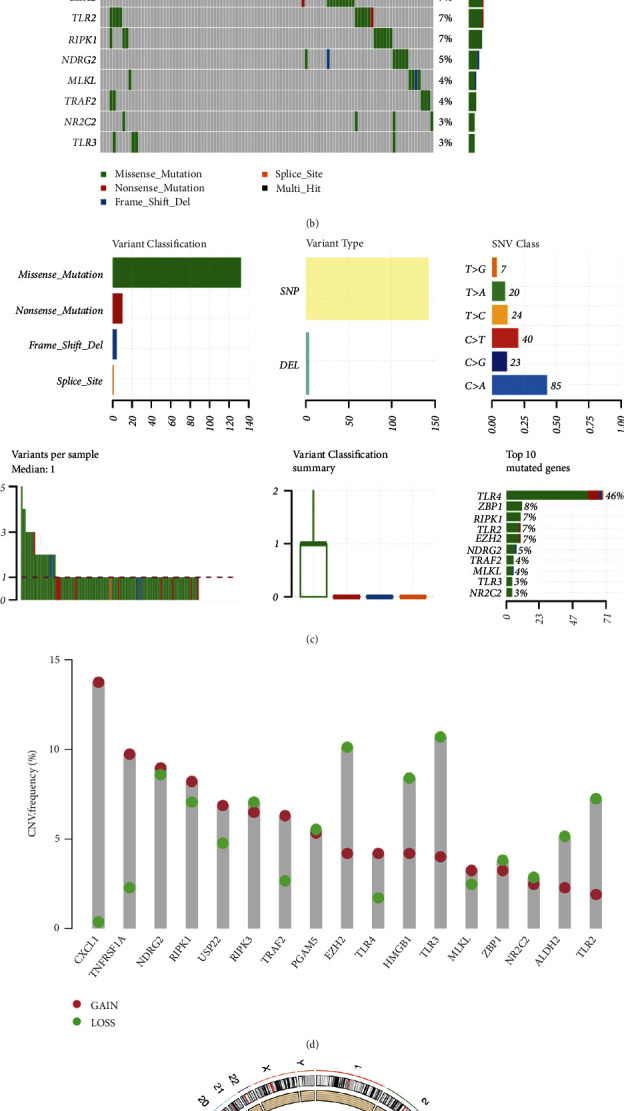
Expression and genetic variation landscape of necroptosis-related genes in LUAD. (a) The mRNA level of 17 necroptosis-related genes in LUAD. (b), and (c) The mutation frequency and classification of 17 necroptosis-related genes in LUAD. (d) The CNV frequency of 17 necroptosis-related genes in LUAD. The height of the column represented the alteration frequency. (e) The location on chromosomes of CNV of 17 necroptosis-related genes. ∗∗*P* < 0.01, ∗∗∗*P* < 0.001.

Supplementary Figure S1 revealed the work flow of the current study.  Figure 1(a) displays the expression landscape, revealing that downregulation of most of necroptosis-related genes were shown in LUAD versus lung tissues while the level of PGAM5, HMGB1, TRAF2, EZH2 was raised (all *p* < 0.05). Figures 1(b) and 1(c) showed the genetic landscape of necroptosis-related gene in LUAD. To be more specific, 64.59% (106/138) of LUAD cases presented with genetic mutations (Figure 1(b)). In terms of mutation rate, TLR4 ranked first, followed by ZBP1 (Figure 1(b)). Missense mutations ranked highest, and C > A was the most common Single Nucleotide Variant (SNV) type (Figure 1(c)). For CNV analysis, the result indicated widespread copy number amplification of CXCL1, TNFRSF1A, NDRG2, RIPK1, USP22, TRAF2, TLR4, and MLKL, as well as widespread CNV deletion of RIPK3, PGAM5, EZH2, HMGB1, TLR3, ZBP1, NR2C2, ALDH2, and TLR2 (Figure 1(d)). The location of CNV alteration of necroptosis-related genes on chromosomes was showed in Figure 1(e).

### 3.2. GO and KEGG Analysis

We then further confirmed whether these genes were associated with necroptosis in LUAD by performing GO and KEGG pathways analysis. Accordingly, these genes were widely associated with programmed necrotic cell death, necrotic cell death, necroptotic process and NF-kappa B signaling, membrane raft, CD40 receptor complex, transcription coregulator activity, cytokine binding, and Tumor necrosis factor (TNF) receptor superfamily binding in GO analysis (Supplementary Figure 2(a)). Furthermore, these necroptosis-related genes showed widespread association with necroptosis, TNF signaling pathway, NF-kappa B signaling pathway, Nucleotide oligomerization domain (NOD)-like receptor signaling pathway, and apoptosis in KEGG pathway analysis (Supplementary Figure 2(b)).

### 3.3. Identification of Two Clusters Based on Necroptosis-Related Genes in LUAD

**Figure 2:**
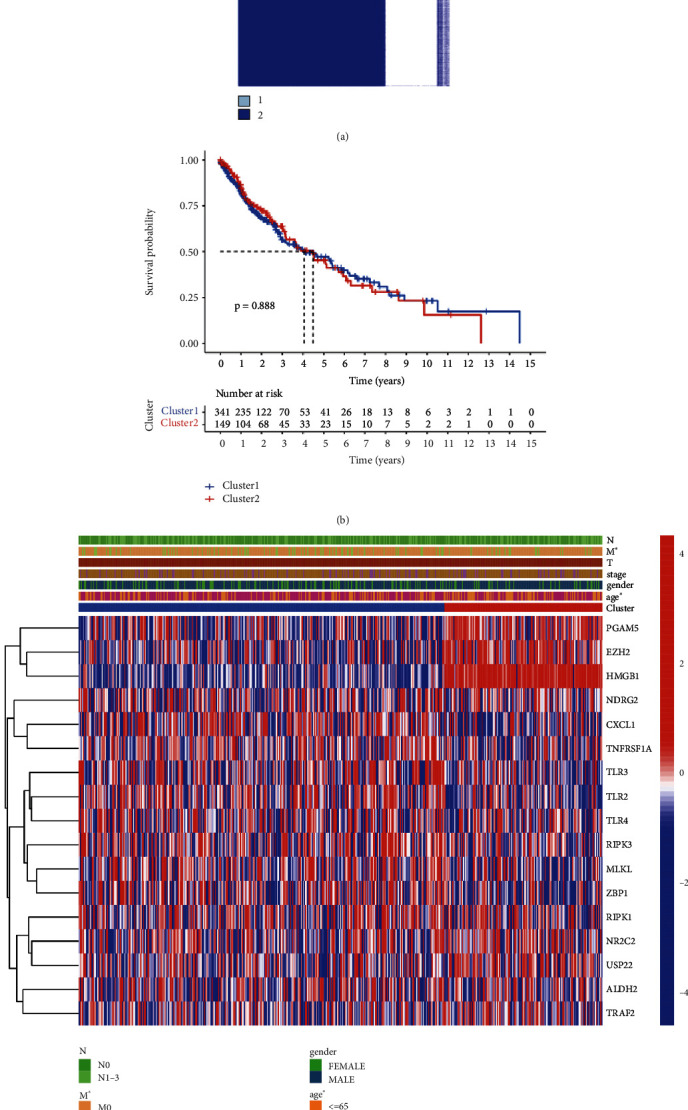
Consensus clustering identified two clusters of LUAD. (a) Consensus clustering matrix about two clusters of LUAD. (b) Overall survival curve in two clusters of LUAD. (c) Heatmap revealed the difference in clinicopathologic features between the two clusters. ∗*P* < 0.05.

**Figure 3:**
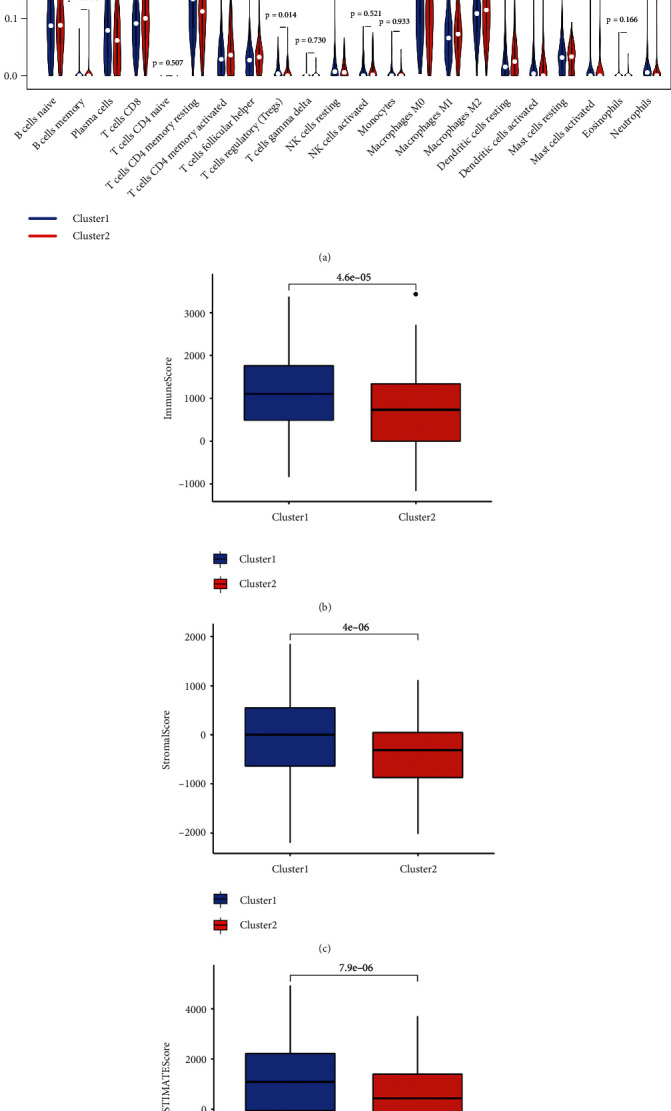
Consensus clustering correlated with distinct immune infiltration in LUAD. (a) The immune cell infiltration landscape in two clusters of LUAD. (b), (c), and (d) Immunoscore, StromaScore, and ESTIMATEScore in two clusters of LUAD.

To clarify whether these LUAD patients can be divided into multiple subgroups to achieve precise treatment for patients, we then performed consensus clustering analysis. LUAD patients were differentiated using consensus clustering based on gene patterns. And two clusters (cluster 1/2) were suggested as the optimal clustering stability based on the similarity (Figure 2(a)). No significant difference was generated in OS rate between these two clusters in LUAD (Figure 2(b), *p* = 0.888). Interestingly, cluster 1 showed distinctly different age and pM stage with cluster 2 (Figure 2(c), *p* < 0.05). In Figure 3(a), the immune cell infiltration landscape was shown for two clusters of LUAD, with cluster 1 correlated with high abundance of plasma cells (*p* = 0.008), CD4 memory resting T cells (*p* = 0.007), Tresgs (*p* = 0.014), and neutrophils (*p* < 0.001) while cluster 1 was correlated with high abundance of CD8 T cells (*p* = 0.024) and follicular helper T cells (*p* = 0.046) in LUAD. Moreover, the data suggested a higher Immunoscore (*p* = 4.6 × 10^−5^), StromaScore (*p* = 4 × 10^−6^), and ESTIMATEScore (*p* = 7.9 × 10^−6^) in cluster 1 versus cluster 2 (Figures 3(b), 3(c), and 3(d)).

### 3.4. A Prognostic Signature Based on Necroptosis-Related Genes

**Figure 4:**
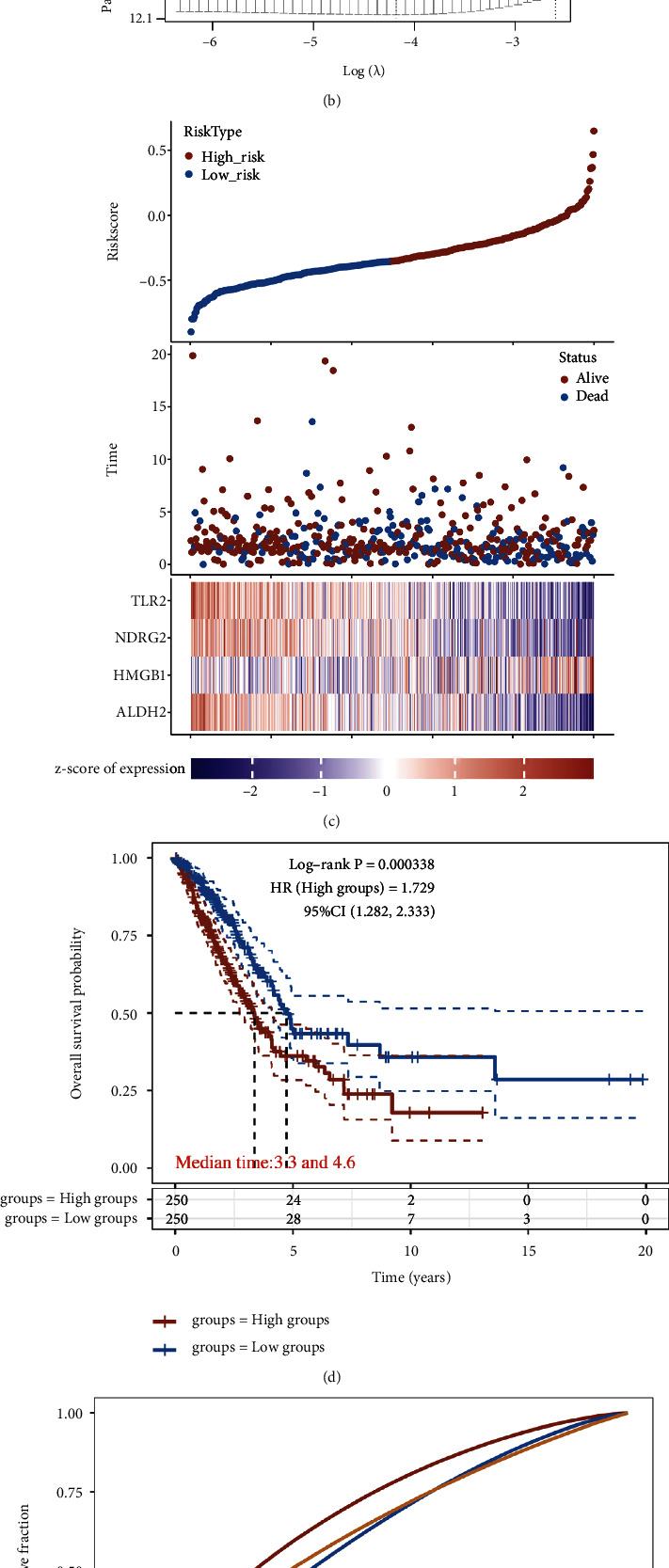
Construction of a necroptosis-related prognostic gene signature. (a), and (b) The coefficient and partial likelihood deviance of prognostic signature. (c) Risk score distribution survival status of patients and gene expression in prognostic signature. (d) and (e) Overall survival curve in patients with high/low risk and the ROC curve evaluated the predictive significance in 1-year, 3-year, and 5-year of LUAD patients.

Prognosis analysis demonstrated that high level of ALDH2, NDRG2, TLR2, TLR4, and low HMGB1 level had a better OS rate in LUAD (Supplementary Figures 3(a), 3(b), 3(c), 3(d), and 3(e)) and Supplementary Table 2). Based on these five necroptosis-related prognostic genes, we then developed a prognostic gene signature with LASSO Cox regression analysis. The coefficients of each LUAD case was calculated with the followed computational equation: risk score = sum of coefficients × the expression of necroptosis-related genes. Finally, based on the result of LASSO Cox regression analysis, the best module was obtained. TLR4 was ejected, and the risk score of patients was calculated by including four other genes in this prognostic signature (Riskscore = (−0.1017) × ALDH2 expression + (0.1559) × HMGB1 expression + (−0.0698) × NDRG2 expression + (−0.0845) × TLR2 expression). The coefficient and partial likelihood deviance of prognostic signature were shown in Figures 4(a) and 4(b). LUAD cohort was divided into high- and low-risk group, and the riskscore, survival status of patients, and gene expression were shown in Figure 4(c). Compared with low-risk group, high-risk group had a worse OS rate (*p* = 0.000338) with the area under the curves in 1-year, 3-year, and 5-year periods being 0.684, 0.592, and 0.584, respectively (Figures 4(d) and 4(e)).

### 3.5. Predictive Nomogram Based on Prognostic Signature and Clinical Characters

**Figure 5:**
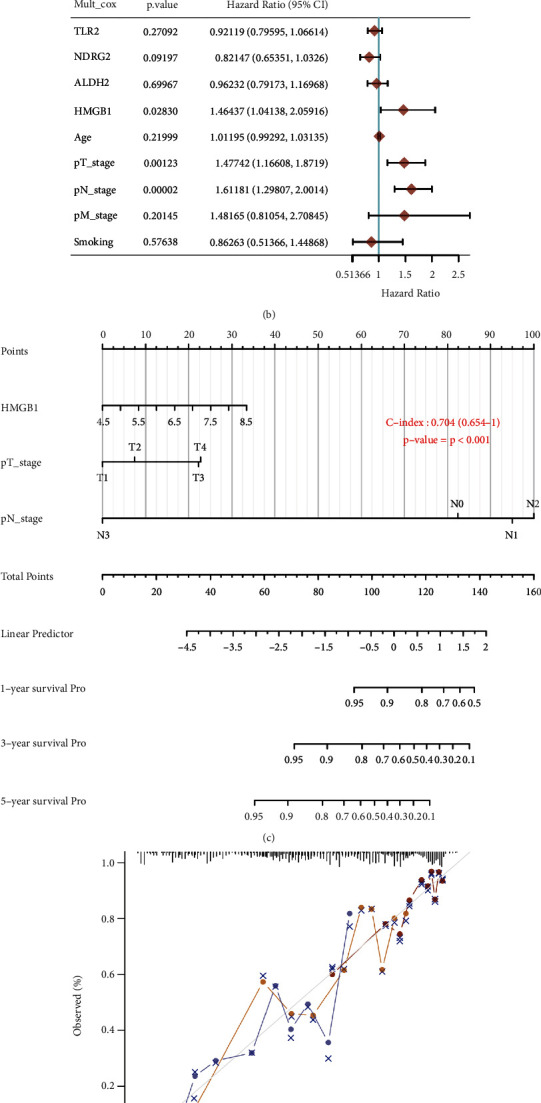
Construction of predictive nomogram. (a) and b) Univariate and multivariate Cox regression considering clinical parameters and necroptosis-related prognostic gene signature. (c) and (d) Nomogram to predict the 1-year, 3-year, and 5-year overall survival of LUAD patients. Calibration curve for the overall survival nomogram model in the discovery group. A dashed diagonal line represents the ideal nomogram. The word “p” means pathological.

Considering clinical characters and above four necroptosis-related prognostic genes, we identified HMGB1, pT stage, and pN stage as independent factors impacting on LUAD patients' prognosis in further analysis (univariate and multivariate analysis) (Figures 5(a) and 5(b)). These factors were used to construct a predictive nomogram to predict survival probability, which showed a good prediction ability (Figures 5(c) and 5(d)).

### 3.6. Individual Analysis of Necroptosis-Related Prognostic Signature

**Figure 6:**
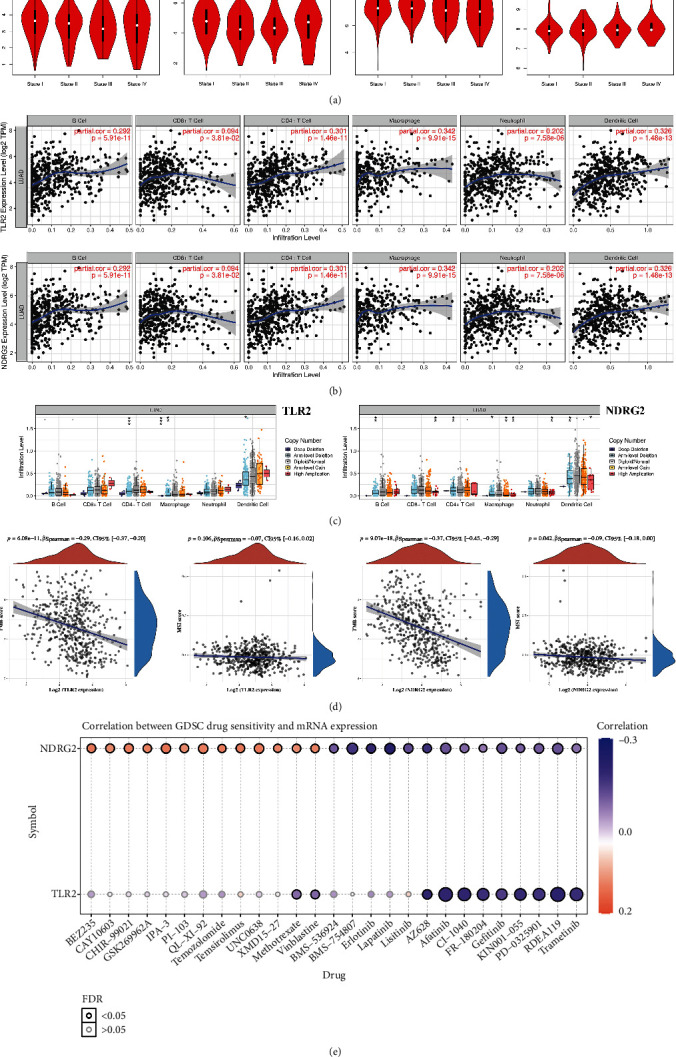
Necroptosis-related genes correlated with pathological stage, immune infiltration, and drug resistance in LUAD. (a) The correlation between pathological stage and TLR2, NDRG2, ALDH2, and HMGB1 in LUAD. (b) The correlation between immune cell infiltration and TLR2/NDRG2 in LUAD. (c) The correlation between copy number alteration of TLR2/NDRG2 and immune cell infiltration in LUAD. (d) The correlation between TLR2/NDRG2 expression and TMB score as well as MSI score in LUAD. (e) The correlation between TLR2/NDRG2 expression and GDSC drug sensitivity. TMB, tumor mutation burden; MSI, microsatellite instability; GDSC, Genomics of Drug Sensitivity in Cancer.

As shown in Figure 6(a), a noteworthy correlation was obtained between pathological stage and TLR2 expression (*p* = 0.0277) and NDRG2 expression (*p* = 0.00581), suggesting that TLR2 and NDRG2 may be correlated with the progression of LUAD. And we select TLR2 and NDRG2 for further study. Previous study had suggested that immune infiltration was involved in tumor development and progression in LUAD [31]. The current result demonstrated a positive correlation between the expression of TLR2 and NDRG2 and the immune infiltration level of B cells, CD8+ T cells, CD4+ T cells, macrophage, neutrophils, and dendritic cells (Figure 6(b), all *p* < 0.05). Moreover, some somatic copy number alterations of TLR2 and NDRG2 could inhibit immune cell infiltration level (Figure 6(c)). TMB and MSI were suggested as predictive marker for cancer immunotherapy, including lung cancer [32–34]. Interestingly, the expression of TLR2 and NDRG2 showed significant correlation with TMB score (*p* = 6.08 × 10^−11^) and MSI score (*p* = 9.07 × 10^−18^) (Figure 6(c)). In MSI analysis, MSI score decreased as NDRG2 expression increased (*p* = 0.042, Figure 6(d)). To identify cancer immunotherapy target, a vital way is to evaluate the correlation between gene expression and existed drug targets. In order to clarify whether TLR2 and NDRG2 could serve as potential drug screening targets, we explored the correlation between TLR2 and NDRG2 and existed drug targets in GDSC database. Interestingly, the expression of TLR2 and NDRG2 showed positive or negative correlation with GDSC drug sensitivity, including methotrexate and vinblastine (Figure 6(e)).

### 3.7. Construction of Necroptosis-Related Regulatory Axis

**Figure 7:**
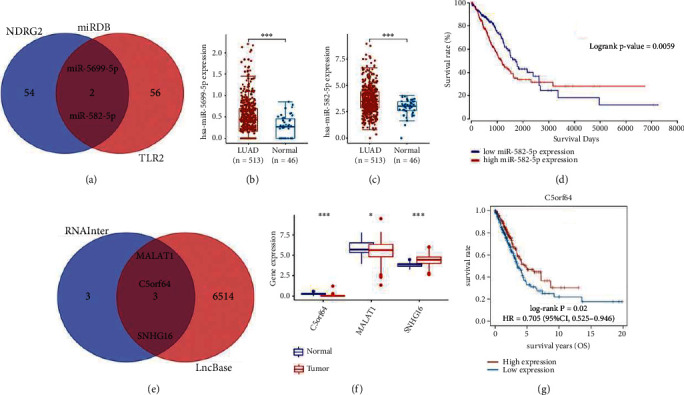
Construction of lncRNA-miRNA-mRNA regulatory axis. (a) miRNA target of TLR2 and NDRG2 predicted by miRDB. The expression of miR-5699-5p (b) and miR-582-5p (c) in LUAD. (d) Overall survival curve in LUAD patients with high and low miR-582-5p expression. (e) lncRNA targets of miR-582-5p predicted by lncBase and RNAIter. (f) The expression of lncRNA C5orf64, MALAT1, and SNHG16 in LUAD. (g) Overall survival curve in LUAD patients with high and low C5orf64 expression. ∗*p* < 0.05, ∗∗*P* < 0.01, ∗∗∗*P* < 0.001.

We then constructed a necroptosis-related regulatory axis to clarify the potential molecular mechanism of TLR2 and NDRG2 in LUAD. Using miRDB, we identified miR-582-5p and miR-5699-5p as the miRNA targets of NDRG2 and TLR2 (Figure 7(a)). Moreover, the expression of miR-582-5p (*p* < 0.001) and miR-5699-5p (*p* < 0.001) were upregulated in LUAD versus lung tissues (Figures 7(b) and 7(c)). OS analysis suggested a poor survival in LUAD patients with high miR-582-5p expression (Figure 7(d), *p* = 0.0059). Accordingly, miR-582-5p may be the most potential miRNA target of NDRG2 and TLR2. To explore its upstream lncRNA targets, we submitted miR-582-5p to RNAInter and lncBase, and the result suggested three lncRNA targets (MALAT1, C5orf64, and SNHG16) interacting to miR-582-5p (Figure 7(e)). Further analyses indicated downregulation of MALAT1, C5orf64, and upregulation of SNHG16 in LUAD versus lung tissues (Figure 7(f), all *p* < 0.05). However, only lncRNA C5orf64 was correlated with OS rate in LUAD (Figure 7(g), *p* = 0.02), suggested C5orf64 as the most promising lncRNA target. Therefore, we identified lncRNA C5orf64/miR-582-5p/NDRG2/TLR2 regulatory axis in the progression in LUAD. This result requires further investigation.

## 4. Discussion

Previous study had suggested the involvement of necroptosis migration and invasion regulation of tumor [35]. The necroptosis mechanism was proposed as an effective way for eradicating cancer cells [36]. The identification of prognostic value and potential regulatory axis of necroptosis-related genes will allow necroptosis to be leveraged for therapeutic benefits and prognosis improvement of LUAD.

To confirm whether these necroptosis-related genes were associated with necroptosis in LUAD, we then performed GO and KEGG pathways analysis. As expected, these necroptosis-related genes showed widespread association with necroptosis, programmed necrotic cell death, necroptotic process, and TNF signaling pathway. These functions or pathways could mediate necroptosis and cancer progression. NF-*κ*B signaling may influenced inflammation and the progression of tumor [37]. Signaling mediated by TNF is also crucial for homeostasis and immunity in mammals [38]. Interestingly, TNF was referred as a key mediator in balancing cell survival and necroptosis [39].

LUAD patients were differentiated using consensus clustering based on gene patterns and we identified two clusters, which showed conspicuous difference in pM stage and immune cell characterization. Cluster 1 of LUAD was linked to high Immunoscore, StromaScore, and ESTIMATEScore and abundant immune cell infiltration, referring to hot tumor [40]. Further moreover, high Immunoscore was significantly correlated with better prognosis in LUAD [40].

Our study also developed a prognostic model based on four prognostic necroptosis-related genes (ALDH2, HMGB1, NDRG2, TLR2), which was highly accurate in predicting LUAD prognosis. Univariate and multivariate analysis identified HMGB1, pT stage, and pN stage as independent factors impacting on LUAD patients' prognosis. Though certain prognostic signatures had been identified for LUAD, our study firstly developed a prognostic model with necroptosis-related markers in LUAD, providing another biomarker in LUAD. A machine learning strategy had constructed and validated a prognostic signature using 12 immune-related genes for LUAD [41]. Another prognostic signature showed good performance in predicting prognosis and reflecting tumor immune microenvironment in LUAD [42]. Jin et al. also constructed a 7-lncRNA prognosis signature and a predictive nomogram in LUAD [43].

The result of our study identified a lncRNA C5orf64/miR-582-5p/NDRG2/TLR2 regulatory axis in the progression in LUAD. Interestingly, previous study demonstrated lncRNA C5orf64 as a novel biomarker associated with tumor microenvironment and mutation pattern remodeling in LUAD [44]. Moreover, miR-582-5p served as a prognostic biomarker in LUAD and inhibited tumor cell proliferation and invasion [45]. High mRNA level of TLR2 could accelerate tumor progression in LUAD [46]. NDRG2 acted a prognostic marker in LUAD and associated with depth of invasion, vascular invasion, and better OS [47]. Thus, lncRNA C5orf64/miR-582-5p/NDRG2/TLR2 regulatory axis may be involved in the progression in LUAD. This result requires further investigation.

Our study also had some limitations. Not all of 17 necroptosis-related genes were specific to necroptosis. Moreover, the result of consensus clustering analysis is barely satisfactory. This result requires further investigation.

## 5. Conclusion

The current bioinformatics analysis identified a necroptosis-related prognostic signature for LUAD and their relation to immunity infiltration. This result requires further investigation.

## Data Availability

The analyzed data sets generated during the study are available from the corresponding author on reasonable request.

## Supplementary Materials

Supplementary Materials Supplementary Table 1. The clinical characters of lung adenocarcinoma patients in TCGA cohort. Supplementary Table 2. The result of necroptosis-related genes in prognosis analysis.

Supplementary Materials Supplementary Figure 1. The work flow of the current study.

Supplementary Materials Supplementary Figure 2. The enriched item in gene ontology (a) and Kyoto Encyclopedia of Genes and Genomes analysis (b). BP, biological process; CC, cellular component; MF molecular function.

Supplementary Materials Supplementary Figure 3. The prognostic value of necroptosis-related genes in LUAD. Overall survival curve in LUAD patients with high/low expression of ALDH2 (a), NDRG2 (b), TLR2 (c), TLR4 (d), and HMGB1 (e).

## References

[1] Siegel R. L., Miller K. D., Jemal A. (2020). Cancer statistics. *CA: a Cancer Journal for Clinicians*.

[2] Denisenko T. V., Budkevich I. N., Zhivotovsky B. (2018). Cell death-based treatment of lung adenocarcinoma. *Cell Death & Disease*.

[3] Cui Y., Fang W., Li C. (2019). Development and validation of a novel signature to predict overall survival in “driver gene-negative” lung adenocarcinoma (LUAD): results of a multicenter study. *Clinical Cancer Research*.

[4] Gong Y., Fan Z., Luo G. (2019). The role of necroptosis in cancer biology and therapy. *Molecular Cancer*.

[5] Zhe-Wei S., Li-Sha G., Yue-Chun L. (2018). The role of necroptosis in cardiovascular disease. *Frontiers in Pharmacology*.

[6] Wu M., Xia Y., Wang Y. (2020). Development and validation of an immune-related gene prognostic model for stomach adenocarcinoma. *Bioscience Reports*.

[7] Yuan J., Amin P., Ofengeim D. (2019). Necroptosis and RIPK1-mediated neuroinflammation in CNS diseases. *Nature Reviews. Neuroscience*.

[8] Najafov A., Chen H., Yuan J. (2017). Necroptosis and cancer. *Trends Cancer*.

[9] Park J. E., Lee J. H., Lee S. Y. (2020). Expression of key regulatory genes in necroptosis and its effect on the prognosis in non-small cell lung cancer. *Journal of Cancer*.

[10] Zhang Z., Xie G., Liang L. (2018). RIPK3-mediated necroptosis and neutrophil infiltration are associated with poor prognosis in patients with alcoholic cirrhosis. *Journal of Immunology Research*.

[11] Chen F., Yang J., Fang M., Wu Y., Su D., Sheng Y. (2022). Necroptosis-related lncRNA to establish novel prognostic signature and predict the immunotherapy response in breast cancer. *Journal of Clinical Laboratory Analysis*.

[12] Wang N., Liu D. (2021). Identification and validation a necroptosis-related prognostic signature and associated regulatory Axis in stomach adenocarcinoma. *Oncotargets and Therapy*.

[13] Zhang W., Cao W., Tong Z. (2022). Identification and validation of a novel necroptosis-related prognostic signature in cervical squamous cell carcinoma and endocervical adenocarcinoma. *Frontiers in Oncology*.

[14] Choi M. E., Price D. R., Ryter S. W., Choi A. M. K. (2019). Necroptosis: a crucial pathogenic mediator of human disease. *JCI Insight*.

[15] Malireddi R. K. S., Kesavardhana S., Kanneganti T. D. (2019). ZBP1 and TAK1: master regulators of NLRP3 inflammasome/pyroptosis, apoptosis, and necroptosis (PAN-optosis). *Frontiers in Cellular and Infection Microbiology*.

[16] Xia X., Lei L., Wang S., Hu J., Zhang G. (2020). Necroptosis and its role in infectious diseases. *Apoptosis*.

[17] Cheng M., Lin N., Dong D., Ma J., Su J., Sun L. (2021). PGAM5: a crucial role in mitochondrial dynamics and programmed cell death. *European Journal of Cell Biology*.

[18] Wen S., Li X., Ling Y. (2020). HMGB1-associated necroptosis and Kupffer cells M1 polarization underlies remote liver injury induced by intestinal ischemia/reperfusion in rats. *The FASEB Journal*.

[19] Zhu J., Yang L. K., Wang Q. H. (2020). NDRG2 attenuates ischemia-induced astrocyte necroptosis via the repression of RIPK1. *Molecular Medicine Reports*.

[20] Lou X., Zhu H., Ning L. (2019). EZH2 regulates intestinal inflammation and necroptosis through the JNK signaling pathway in intestinal epithelial cells. *Digestive Diseases and Sciences*.

[21] Petersen S. L., Chen T. T., Lawrence D. A., Marsters S. A., Gonzalvez F., Ashkenazi A. (2015). TRAF2 is a biologically important necroptosis suppressor. *Cell Death and Differentiation*.

[22] Roedig J., Kowald L., Juretschke T. (2021). USP22 controls necroptosis by regulating receptor-interacting protein kinase 3 ubiquitination. *EMBO Reports*.

[23] Ritchie M. E., Phipson B., Wu D. (2015). Limma powers differential expression analyses for RNA-sequencing and microarray studies. *Nucleic Acids Research*.

[24] Mayakonda A., Lin D. C., Assenov Y., Plass C., Koeffler H. P. (2018). Maftools: efficient and comprehensive analysis of somatic variants in cancer. *Genome Research*.

[25] Zhang H., Meltzer P., Davis S. (2013). RCircos: an R package for Circos 2D track plots. *BMC Bioinformatics*.

[26] Ito K., Murphy D. (2013). Application of ggplot2 to Pharmacometric graphics. *CPT: Pharmacometrics & Systems Pharmacology*.

[27] Wilkerson M. D., Hayes D. N. (2010). ConsensusClusterPlus: a class discovery tool with confidence assessments and item tracking. *Bioinformatics*.

[28] Chen Y., Wang X. (2020). miRDB: an online database for prediction of functional microRNA targets. *Nucleic Acids Research*.

[29] Karagkouni D., Paraskevopoulou M. D., Tastsoglou S. (2020). DIANA-LncBase v3: indexing experimentally supported miRNA targets on non-coding transcripts. *Nucleic Acids Research*.

[30] Lin Y., Liu T., Cui T. (2020). RNAInter in 2020: RNA interactome repository with increased coverage and annotation. *Nucleic Acids Research*.

[31] Bremnes R. M., Busund L. T., Kilvær T. L. (2016). The role of tumor-infiltrating lymphocytes in development, progression, and prognosis of non-small cell lung cancer. *Journal of Thoracic Oncology*.

[32] Liu L., Bai X., Wang J. (2019). Combination of TMB and CNA stratifies prognostic and predictive responses to immunotherapy across metastatic cancer. *Clinical Cancer Research*.

[33] Samstein R. M., Lee C. H., Shoushtari A. N. (2019). Tumor mutational load predicts survival after immunotherapy across multiple cancer types. *Nature Genetics*.

[34] Chang L., Chang M., Chang H. M., Chang F. (2018). Microsatellite instability: a predictive biomarker for cancer immunotherapy. *Applied Immunohistochemistry & Molecular Morphology*.

[35] Ando Y., Ohuchida K., Otsubo Y. (2020). Necroptosis in pancreatic cancer promotes cancer cell migration and invasion by release of CXCL5. *PLoS One*.

[36] Philipp S., Sosna J., Adam D. (2016). Cancer and necroptosis: friend or foe?. *Cellular and Molecular Life Sciences*.

[37] Hoesel B., Schmid J. A. (2013). The complexity of NF-*κ*B signaling in inflammation and cancer. *Molecular Cancer*.

[38] Blaser H., Dostert C., Mak T. W., Brenner D. (2016). TNF and ROS crosstalk in inflammation. *Trends in Cell Biology*.

[39] Grootjans S., Vanden Berghe T., Vandenabeele P. (2017). Initiation and execution mechanisms of necroptosis: an overview. *Cell Death and Differentiation*.

[40] Sun S., Guo W., Wang Z. (2020). Development and validation of an immune-related prognostic signature in lung adenocarcinoma. *Cancer Medicine*.

[41] Xue L., Bi G., Zhan C., Zhang Y., Yuan Y., Fan H. (2020). Development and validation of a 12-gene immune relevant prognostic signature for lung adenocarcinoma through machine learning strategies. *Frontiers in Oncology*.

[42] Song Q., Shang J., Yang Z. (2019). Identification of an immune signature predicting prognosis risk of patients in lung adenocarcinoma. *Journal of Translational Medicine*.

[43] Jin D., Song Y., Chen Y., Zhang P. (2020). Identification of a seven-lncRNA immune risk signature and construction of a predictive nomogram for lung adenocarcinoma. *BioMed Research International*.

[44] Pang Z., Chen X., Wang Y. (2021). Long non-coding RNA C5orf64 is a potential indicator for tumor microenvironment and mutation pattern remodeling in lung adenocarcinoma. *Genomics*.

[45] Wang L. L., Zhang M. (2018). miR-582-5p is a potential prognostic marker in human non-small cell lung cancer and functions as a tumor suppressor by targeting MAP3K2. *European Review for Medical and Pharmacological Sciences*.

[46] Li J., Zhang C., Gong S. (2019). A nanoscale photothermal agent based on a metal-organic coordination polymer as a drug-loading framework for effective combination therapy. *Acta Biomaterialia*.

[47] Li S. J., Wang W. Y., Li B. (2013). Expression of NDRG2 in human lung cancer and its correlation with prognosis. *Medical Oncology*.

