# Supplementary material for: Identification of a Necroptosis-Related Prognostic Signature and Associated Regulatory Axis in Lung Adenocarcinoma
Source: Int J Genomics. 2023 Feb 22;2023:8766311. doi: 10.1155/2023/8766311 (PMC10643042; doi:10.1155/2023/8766311)
Supplement: Supplementary Materials — Supplementary Table 1. The clinical characters of lung adenocarcinoma patients in TCGA cohort. Supplementary Table 2. The result of necroptosis-related genes in prognosis analysis. [file 8766311.f1.docx]

Supplementary Table 1. The clinical characters of lung adenocarcinoma patients in TCGA cohort.

| Clinical characters | Number |
| --- | --- |
| Gender  Male  Female | 222  264 |
| Age (years, x ± s) | 65.01 ± 10.04 |
| TNM stage  I  II  III  IV  Unknown | 263  111  79  25  8 |
| T  T1  T2  T3  T4  Unknown | 163  260  41  19  3 |
| N  N0  N1  N2  N3  Nx  Unknown | 312  90  70  2  11  1 |
| M  M0  M1  Mx  Unknown | 333  24  125  4 |

Supplementary Table 2. The result of necroptosis‑related genes in prognosis analysis.

| Genes | p-value | HR | Low 95%CI | High 95%CI |
| --- | --- | --- | --- | --- |
| ALDH2 | 0.00758283 | 0.67041495 | 0.49988815 | 0.89911353 |
| EZH2 | 0.57092621 | 1.08748455 | 0.81367146 | 1.45343999 |
| HMGB1 | 0.02546431 | 1.39755235 | 1.04195273 | 1.87451169 |
| MLKL | 0.15188709 | 1.23775641 | 0.92454014 | 1.65708428 |
| NDRG2 | 0.00492592 | 0.65646373 | 0.48955256 | 0.88028266 |
| NR2C2 | 0.35945563 | 0.87189676 | 0.65033067 | 1.16894987 |
| PGAM5 | 0.38801254 | 1.13634971 | 0.85010306 | 1.51898132 |
| RIPK1 | 0.86883518 | 0.97580033 | 0.729609 | 1.30506379 |
| RIPK3 | 0.16540432 | 0.81298037 | 0.60677239 | 1.0892669 |
| TLR2 | 0.03558248 | 0.72979685 | 0.54403576 | 0.97898611 |
| TLR3 | 0.21463925 | 1.20238895 | 0.89870532 | 1.60869103 |
| TLR4 | 0.01761763 | 0.69912005 | 0.5202241 | 0.93953519 |
| TRAF2 | 0.17659047 | 1.2221061 | 0.91362139 | 1.63475081 |
|  |  |  |  |  |
