# Supplementary figures and images for: Identification of a Necroptosis-Related Prognostic Signature and Associated Regulatory Axis in Lung Adenocarcinoma
Source: Int J Genomics. 2023 Feb 22;2023:8766311. doi: 10.1155/2023/8766311 (PMC10643042; doi:10.1155/2023/8766311)

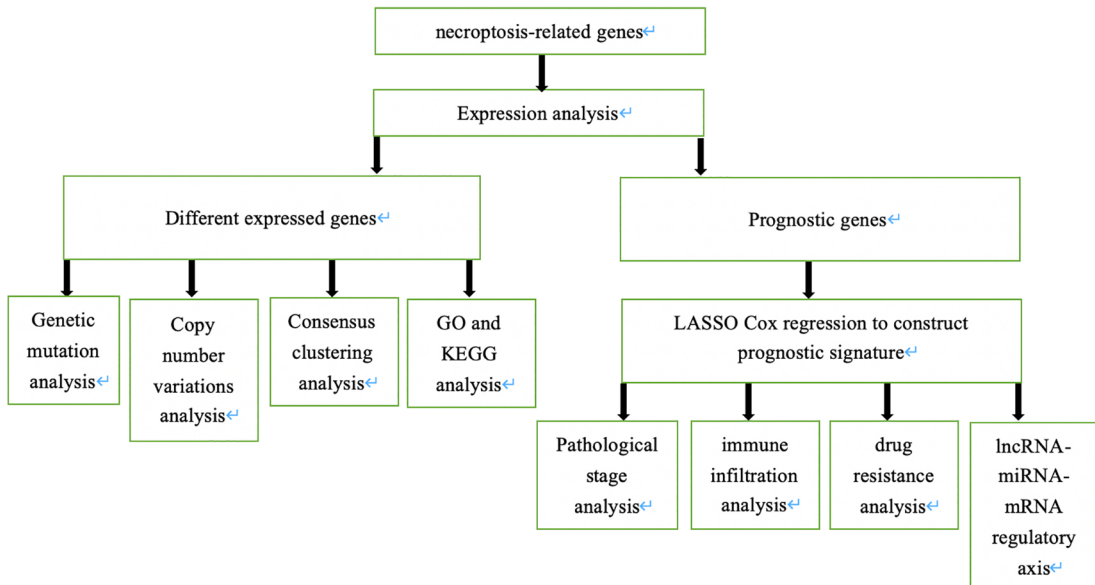

Supplement: Supplementary Materials — Supplementary Figure 1. The work flow of the current study. [file 8766311.f2.pdf]

A

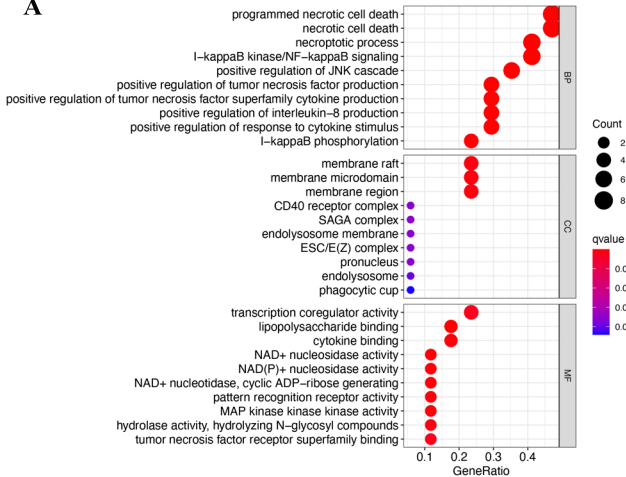

B

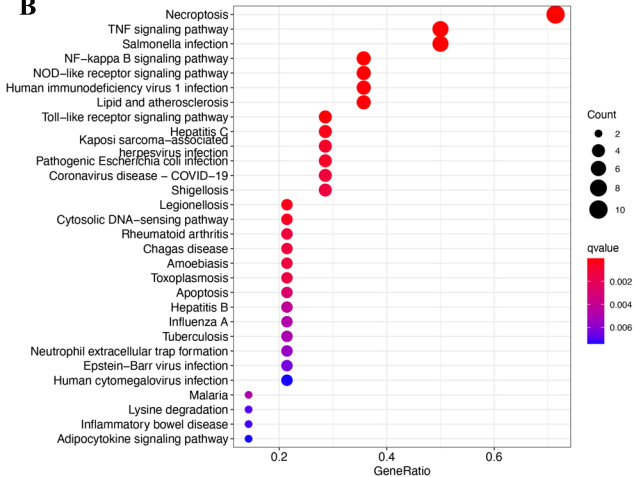

Supplement: Supplementary Materials — Supplementary Figure 2. The enriched item in gene ontology (a) and Kyoto Encyclopedia of Genes and Genomes analysis (b). BP, biological process; CC, cellular component; MF molecular function. [file 8766311.f3.pdf]

**A****ALDH2**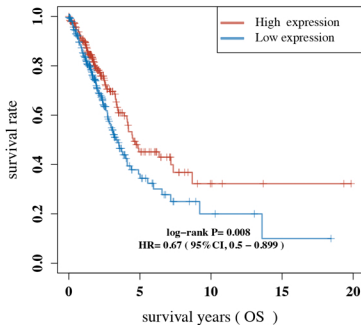**B****NDRG2**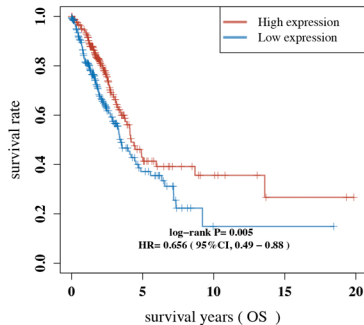**C****TLR2**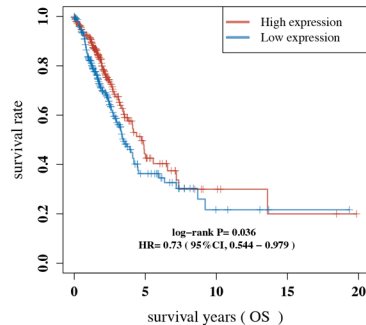**D****TLR4**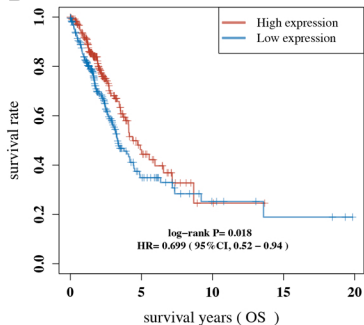**E****HMGB1**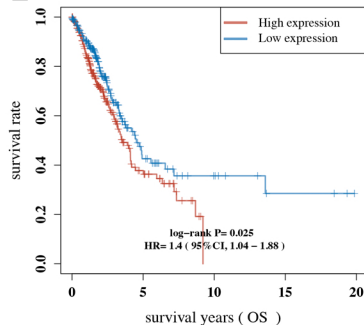

Supplement: Supplementary Materials — Supplementary Figure 3. The prognostic value of necroptosis-related genes in LUAD. Overall survival curve in LUAD patients with high/low expression of ALDH2 (a), NDRG2 (b), TLR2 (c), TLR4 (d), and HMGB1 (e). [file 8766311.f4.pdf]
